# Early prediction of pediatric asthma in the Canadian Healthy Infant Longitudinal Development (CHILD) birth cohort using machine learning

**DOI:** 10.1038/s41390-023-02988-2

**Published:** 2024-01-11

**Authors:** Ping He, Theo J. Moraes, Darlene Dai, Myrtha E. Reyna-Vargas, Ruixue Dai, Piush Mandhane, Elinor Simons, Meghan B. Azad, Courtney Hoskinson, Charisse Petersen, Kate L. Del Bel, Stuart E. Turvey, Padmaja Subbarao, Anna Goldenberg, Lauren Erdman

**Affiliations:** 1 Center for Computational Medicine, The Hospital for Sick Children, Toronto, ON Canada; 2 Translational Medicine Program, The Hospital for Sick Children, Toronto, ON Canada; 3 Department of Pediatrics, BC Children’s Hospital, University of British Columbia, Vancouver, BC Canada; 4 University of Alberta, Edmonton, AB Canada; 5 Department of Pediatrics & Child Health, University of Manitoba, Winnipeg, MB Canada; 6 Department of Microbiology and Immunology, University of British Columbia, Vancouver, BC Canada; 7 Department of Computer Science, University of Toronto, Toronto, ON Canada; 8 Department of Genetics and Genome Biology, The Hospital for Sick Children, Toronto, ON Canada; 9 Vector Institute, Toronto, ON Canada; 10 CIFAR, Toronto, ON Canada; 11 James M. Anderson Center for Health Centers Excellence, Cincinnati Children’s Hospital Medical Center, Cincinnati, OH USA

## Abstract

**Background:**

Early identification of children at risk of asthma can have significant clinical implications for effective intervention and treatment. This study aims to disentangle the relative timing and importance of early markers of asthma.

**Methods:**

Using the CHILD Cohort Study, 132 variables measured in 1754 multi-ethnic children were included in the analysis for asthma prediction. Data up to 4 years of age was used in multiple machine learning models to predict physician-diagnosed asthma at age 5 years. Both predictive performance and variable importance was assessed in these models.

**Results:**

Early-life data (≤1 year) has limited predictive ability for physician-diagnosed asthma at age 5 years (area under the precision-recall curve (AUPRC) < 0.35). The earliest reliable prediction of asthma is achieved at age 3 years, (area under the receiver-operator curve (AUROC) > 0.90) and (AUPRC > 0.80). Maternal asthma, antibiotic exposure, and lower respiratory tract infections remained highly predictive throughout childhood. Wheezing status and atopy are the most important predictors of early childhood asthma from among the factors included in this study.

**Conclusions:**

Childhood asthma is predictable from non-biological measurements from the age of 3 years, primarily using parental asthma and patient history of wheezing, atopy, antibiotic exposure, and lower respiratory tract infections.

**Impact:**

- Machine learning models can predict physician-diagnosed asthma in early childhood (AUROC > 0.90 and AUPRC > 0.80) using ≥3 years of non-biological and non-genetic information, whereas prediction with the same patient information available before 1 year of age is challenging.
- Wheezing, atopy, antibiotic exposure, lower respiratory tract infections, and the child’s mother having asthma were the strongest early markers of 5-year asthma diagnosis, suggesting an opportunity for earlier diagnosis and intervention and focused assessment of patients at risk for asthma, with an evolving risk stratification over time.

## Introduction

Asthma is a prevalent chronic disease in children, affecting approximately 4.1 million children worldwide,^1^ with no cure currently available. Currently, the lack of accurate tools to predict which children are at risk of developing lifelong asthma presents a significant challenge in the management of childhood asthma. Precise detection of persistent childhood asthma before 5 years of age is difficult, leading to both over-treatment and under-treatment of preschool children.^2^ Therefore, a clinically adaptable predictive tool for estimating a child’s risk of developing asthma by school age could prove invaluable to children susceptible to asthma and physicians providing early asthma care for preschool children. Such a tool would help identify high-risk children early on and provide appropriate interventions, avoiding unnecessary treatment while ensuring that high-risk children receive early and effective treatment to prevent or manage their symptoms.

In recent years, machine learning (ML) approaches have gained popularity in healthcare research due to their ability to integrate heterogeneous data, handle complex interactions between variables, and identify patterns from large datasets. Compared to traditional regression-based approaches, ML models have the potential to identify predictors that may have been overlooked and capture nonlinear and complex interactions between variables, particularly for disease prediction.^3–5^

In the context of asthma, previous studies have applied ML methods to predict asthma diagnosis or identify different subtypes of asthma, known as endotypes. These studies have shown promising results, demonstrating the potential of ML models in predicting asthma. For instance, a Support Vector Machine (SVM) model was used to predict asthma at age 10 years by age 4 years (AUROC of 0.82) and differentiate between allergic and non-allergic asthma (AUROC of 0.79).^6,7^

However, the studies mentioned above had limitations such as small sample sizes and a lack of ethnic diversity among study populations, which restricted the generalizability of their findings to larger populations. Despite these limitations, the potential of ML in predicting on-going childhood asthma diagnosis and identifying endotypes suggests a pressing need for developing a clinically adaptable predictive tool for childhood asthma that can provide appropriate interventions for high-risk children.

To address this gap, we utilized the CHILD Study, a longitudinal study conducted across four Canadian provinces, to predict childhood asthma in a large, multi-ethnic population. Our study collected data on family medical history, early-life clinical and environmental factors for children up to 4 years of age, and leveraged five different types of ML models and two sets of ensemble algorithms to predict asthma diagnosis at 5 years of age. Our primary objective was to identify the earliest time point for accurate asthma prediction and to determine the relative importance of each predictor over time, thereby laying the foundation for the development of a clinically applicable asthma prediction tool that can aid in clinical identification and treatment of high-risk children.

## Methods

### Study design

This study utilized data from the CHILD Cohort Study, one of the largest ongoing longitudinal birth cohort studies in Canada that gathered data on asthma and allergy research from mid-pregnancy through childhood. The study recruited pregnant women from multiple sites across four Canadian provinces between 2008 and 2012.^8,9^ The study was approved centrally by the Hamilton Integrated Research Ethics Board (HiREB #07-2929).

For this study, only children with complete CHILD questionnaires and physician-diagnosed asthma outcomes from clinical visits at age 5 years were included. The dataset was then stratified by asthma status and randomly split into two subsets: a training dataset (85%) used for training and tuning ML models, and a holdout dataset (15%) for assessing the models’ performance. The training dataset comprised data from 1484 children, including 1395 non-asthmatic and 89 asthmatic children, while the holdout dataset comprised data from 270 children, with 250 non-asthmatic and 20 asthmatic children. The test set was then set aside while the training set was exclusively used for model tuning and feature selection.

**Fig. 1:**
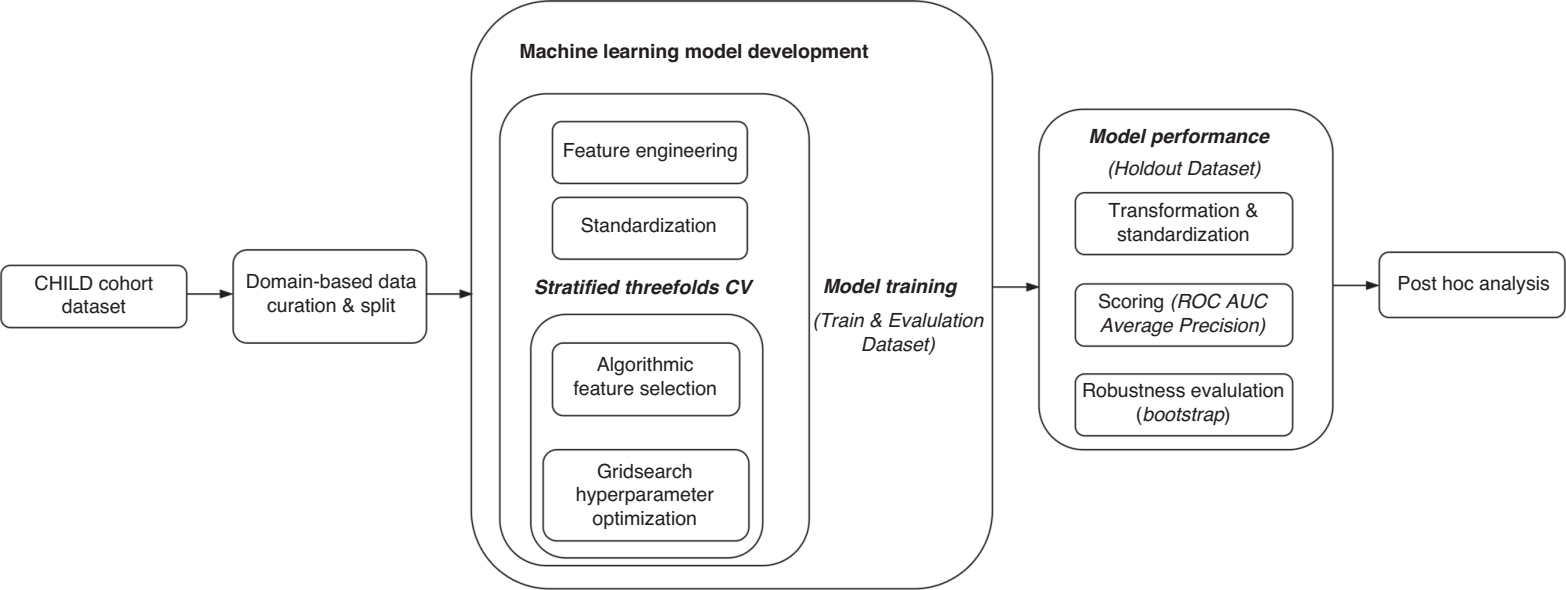
ML workflow diagram. All data were obtained from the CHILD Cohort Study. After the collected data was curated, the complete dataset was split into two subsets: (1) Training & Evaluation Dataset and (2) Holdout Dataset. ML models were developed using the training & evaluation dataset. We identified the optimal feature engineering type using trial and error observation. We used the minimal and maximal scaling technique for the standardization of all the input data for the comparability of features. A stratified *K*-fold cross-validation (*K* = 3 in our study) was applied for the processes of algorithmic feature selection and model hyperparameter optimization. After the features and hyperparameters were identified, we used the developed models to observe its performance on the holdout dataset. Thereafter, a bootstrap technique was adopted to observe the range of scoring of model predictive power. Finally, we performed a post hoc analysis on the features’ importance at different time points.

To identify the best ML algorithms for predicting childhood asthma, the study compared the predictive performance of five different models: Logistic Regression, Random Forest, eXtreme Gradient Boost, Decision Tree, and Support Vector Machine. Hyperparameters for each algorithm were tuned using a grid search space for asthmatic data, with threefold cross-validation used for both feature selection and model tuning. After tuning each algorithm with the training dataset, two sets of ensemble methods (voting and stacking algorithms) were generated to determine if combining individual models could improve predictive performance. Last, the final models were applied to the test set to assess their generalization in previously unseen data. The ML workflow is illustrated in Fig. 1.

### Variable inclusion

We used 132 variables from six time points (birth, 6 months, 1 year, 2 years, 3 years, and 4 years) as input for each ML algorithm to predict physician-diagnosed asthma at the 5-year clinic visit. All available longitudinal data with <10% missing observations are included in the model to perform feature selection and longitudinal investigation. Data such as gut microbiome, breastfeeding microbiome, and dust phthalate, were left out of the current study due to low sample sizes in these sets (*n* < 1000), relative to the full dataset (*n* > 1500). The data included for modeling reflected three aspects of the birth cohort: parental information (e.g., maternal and paternal diagnosed asthma, parental allergies, maternal psychological health), children’s clinical information (e.g., anthropometrics, mode of delivery, sex, gestational age, atopy, wheezing status, respiratory infection history), and environmental information (e.g., home environment, breastfeeding history, antibiotic exposure). Supplement Table 1 provides more details on the variables together with the definitions for both outcome and variables used in the study.

### Preprocessing

To prepare the dataset for use with ML algorithms, a series of preprocessing steps were taken, as illustrated in Supplement Fig. 1. Different feature engineering schemes were applied to highlight the importance of each feature, including converting continuous scores into categorical data by binning them into different bands and labeling them based on existing literature (such as Apgar scoring and PSS/CES-D scoring),^10–12^ log-transforming highly dispersed data (e.g., hospital stay duration after birth), and splitting categorical features into several binary features to assess their impact (e.g., mode of delivery). To impute missing values, we used a MissForest (based on random forest)^13^ imputer for correlated features (e.g., weight for age), and the median values were used for uncorrelated values (e.g., number of pregnancies before delivery). For categorical features, the most frequent values were applied to fill in missing values. Highly correlated features with a correlation coefficient of 0.98 or higher were removed to avoid the effects of multicollinearity. Finally, a minimal-maximal scaler was applied to all features to enable feature comparison and interpretation of their importance.

### Feature selection

In view of the high dimensionality, complexity, and collinearity of our dataset, we employed Sequential Feature Floating Selection (SFFS),^14^ an algorithmic feature selection technique, to identify the subset of features that provided the best model performance by sequentially scanning each input variable for predicting asthma and selecting the appropriate subset of features for model building. To investigate the longitudinal importance of each feature in predicting age 5-year asthma, we grouped all input variables at each time point, and at each time point, SFFS was used to identify the optimal subset of features for each type of ML algorithm used in the study. This feature selection process was conducted in a time-sequential, cumulative manner, where the previously optimally selected subset of features, combined with the newly available variables at the next time point, were used as the input feature set for feature selection at the next time point. During the feature selection process, we employed a stratified threefold cross-validation within the training set to assess the averaged model performance given each selected subset of features.

### Model performance evaluation

The area under the receiver-operating characteristic curve (AUROC) is a widely used metric to evaluate the performance of ML models.^6,7^ However, when dealing with imbalanced datasets, studies have shown that the precision-recall curve provides a more informative measure of performance. The area under the precision-recall curve (AUPRC) can be utilized as a summary statistic to evaluate the performance of models on imbalanced datasets. In this study, we used both AUROC and AUPRC to evaluate the predictive performance of each model on the holdout dataset. The holdout dataset was identical for all tests, ensuring that the class imbalance was consistent, allowing for a direct comparison of AUROC and AUPRC values between models and time points.

### Model robustness estimation

To evaluate the reliability of our model performance metrics, we employed a bootstrap method to calculate the 95% confidence interval for each performance measure and ML algorithm employed. We accomplished this by repeatedly resampling our model results 30 times with replacement, utilizing these samples to determine the 2.5th and 97.5th percentile values. We then employed these values as the lower- and upper-confidence bounds, respectively, for the 95% confidence interval.

### Ensemble modeling

Our study also explored the efficacy of two different sets of ensemble algorithms to enhance the predictive performance of the distinctive individual models.^15^ One set consists of voting classifiers, which utilize averaged or weighted probabilities obtained from the individual models to arrive at the final prediction of the ensemble model. The voting technique is aimed at leveraging the collective intelligence of the individual models. The other set is referred to as stacking classifiers, where the predictions of all the individual models serve as new features for a final estimator or meta-estimator. This approach allows us to exploit the strengths of each individual estimator in a non-linear manner.

## Results

### Longitudinal model performance across early childhood stages

**Fig. 2:**
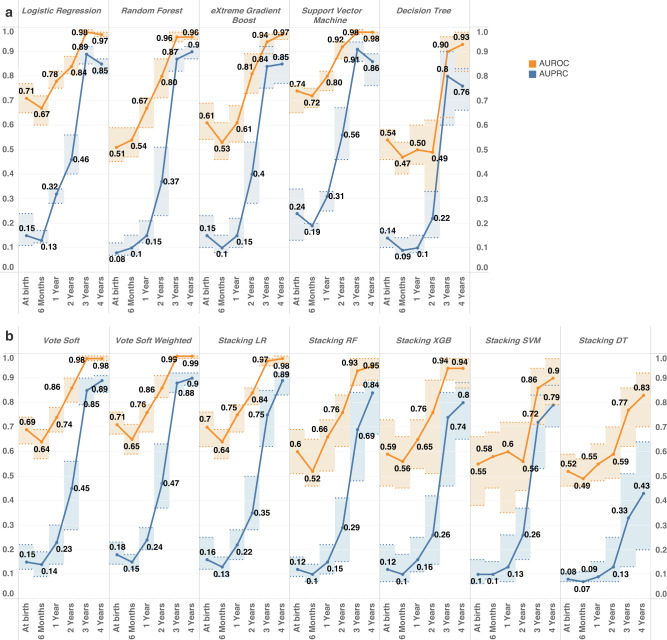
ML model performance for asthma prediction at age 5 years in the CHILD Cohort. **a** Five types of individual ML model predictive performance across six different time points for 5-year physician-diagnosed asthma in the CHILD Cohort. The orange line represents the model performance metric area under receiver operating curve (AUROC), whereas the blue line represents the area under precision recall curve (AUPRC) for the model developed at different time points. The shaded band at each time point represents the 95% confidence interval for the corresponding metrics calculated from 30 iterations of bootstrapped training samples. **b** Ensemble model predictive performance along different time points. The “Soft” in Soft Vote and Soft Weighted means the voting algorithms are ensemble algorithms based on predicted probability outcome rather than binary outcome from individual models. The “LR,” “RF,” “XGB,” “DT,” and “SVM” following the “Stacking” in the naming suggests the final estimator used for the stacking ensemble algorithms. They are Logistic Regression, Random Forest, eXtreme Gradient Boost, Decision Tree, and Support Vector Machine, respectively.

In this study, the predictive performance of five distinct types of ML models was tested using recursively selected variables at six different time points (birth, 6 months, 1 year, 2 years, 3 years, and 4 years), as illustrated in Fig. 2a. The performance of each trained model was assessed using the AUROC and AUPRC metrics, with bootstrapped 95% confidence intervals.

In general, as shown in Fig. 2a, b, both individual and ensemble models exhibited a consistent improvement in performance from early life to 4 years of age. Nonetheless, there was a noticeable decline in performance between birth and 6 months for certain models such as SVM and Decision Tree. This drop in performance, along with the generally poor performance during this period, suggested a considerable degree of uncertainty in predicting asthma during the first year of life. The most significant increase in model predictive power was observed between 2 years and 3 years of age, coinciding with the inclusion of new observation of variables at 3 years of age. At 4 years of age, the predictive power of all models appeared to remain at the same level as that observed at 3 years of age, with a slight decrease in AUPRC for certain models (Logistic Regression and SVM).

Most individual ML models had AUROC scores below 0.75 when using features from before 1 year of age. Performance improved to around 0.80 AUROC with new observations at 2 years of age, and was further enhanced to over 0.90 with the inclusion of new features available at 3 and 4 years of age. The highest AUROC score of 0.99 was achieved at age 4 (Fig. 2a, b). Similarly, AUPRC scores followed a similar pattern, increasing from <0.25 at <1 year of age to >0.8 at 3 and 4 years of age, with the highest AUPRC score of 0.91.

In addition to longitudinal differences, the predictive performance of each type of ML algorithm was assessed. Logistic regression, Random Forest, and SVM achieved their highest predictive power at age 3 years and remained highly predictive at age 4, with SVM even reaching 0.92 AUROC as early as age 2 years. In contrast, Decision Tree models performed the poorest and showed high instability (as seen in Fig. 2a). All ML models, except for Decision Tree models, demonstrated narrower confidence intervals when developed with data up to 3 and 4 years of age, indicating a stronger association between features available at 3 years of age and beyond with asthma diagnosis at age 5 years.

Our adopted ensemble models exhibited a consistent trend in performance over time, with the best performance obtained using features available at 4 years of age for soft-weighted voting algorithms, as illustrated in Fig. 2a, b. Notably, the predictive power of the ensemble models was comparable to that of the individual models. The voting-based ensemble algorithms with 3- and 4-year features demonstrated exceptional performance, achieving AUROCs of 0.98 and 0.99, respectively, with narrower confidence intervals. These models emerged as the best-performing in all our analyses. In contrast, stacking algorithms showed similar or lower performance than the individual models, with decision tree as the mega-estimator exhibiting the lowest and most unstable predictive power.

### Longitudinal feature importance across early childhood stages

To identify the features with the greatest impact on predicting asthma diagnosis at age 5 years, we computed feature importance at each time point using all the ML models trained on the training dataset. We derived feature importance by combining the linear model coefficients, mean decrease in impurity from the tree-based models, and permutation importance for SVM. We then ranked the features based on their overall contributions to our models.

**Fig. 3:**
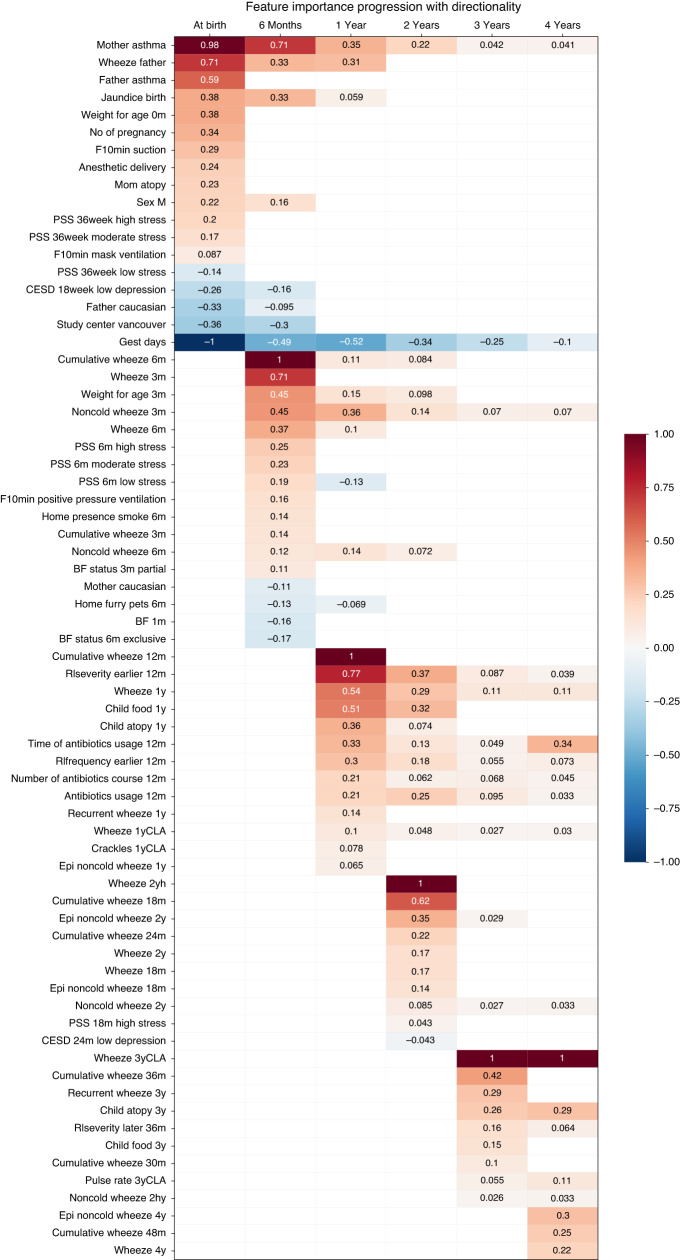
Feature importance progression for asthma prediction across six time points in the CHILD Cohort. At each time point, all the feature importance scores are normalized based on the highest score, which is set to +/−1. The background colors indicate the directionality of the feature—those in red indicate they might be a risk factor (positive association with asthma at age 5 years), those in blue indicate they might have protective effect (negative association with asthma at age 5 years). In the naming of features, “F10min” represents the measures taken within 10 min after a child is delivered. CESD represents “Center for Epidemiologic Studies Depression,” an indicator for mental wellbeing. PSS represents “Psychological Stress Scale.” “BF” represents “Breastfeeding,” “Epi” represents “Episodes,” “RIseverity” represents “the severity of (lower tract) respiratory infections,” whereas the “RIfrequency” represents “the frequency of respiratory infections,” “Cumulative wheeze” refer to the “total number of wheeze,” and “CLA” refer to “clinically assessed by a physician” rather than the rest “self-reported” information.

Our analysis revealed that the top predictors for 5-year asthma diagnosis at birth were parental asthma (maternal or paternal), gestational age, and maternal psychological status during pregnancy (Fig. 3). Other influential features included parental ethnicity (Supplemental Table 2), sex, birth weight, and jaundice. However, many of these features lost importance as data from later time points were included, suggesting they may be proxies for later features or less predictive than features at later time points for 5-year asthma prediction.

At 6 months, children’s wheezing status, particularly cumulative wheezing and wheezing in the absence of a cold, became the most important predictor for 5-year asthma diagnosis. Maternal psychological wellbeing, gestational age, and exclusive breastfeeding at 6 months continued to have a significant effect, while the presence of smoke at home had a negative influence.

By 1 year of age, the child’s atopic status, lower respiratory tract infections, and antibiotic exposures became highly important predictors of asthma diagnosis at age 5 years, along with wheezing status. Parental ethnicity, sex, birth weight, and breastfeeding lost their importance. However, models from birth to 1 year had poor predictive performance (AUROC ≤ 0.8 and AUPRC ≤ 0.35).

At 2 years of age, the most recent wheezing status was the most important predictor, and atopy, respiratory infections, antibiotic exposure, maternal asthma, and gestational days remained significant. Most models had a higher performance (AUROC > 0.80 and AUPRC > 0.40) compared to models from birth to 1 year.

Including data up to age 3 years, wheezing status and atopy at 3 years (wheezing status in the 3-year clinic visit and recurrent wheeze at 36 months of age) were highly predictive for asthma diagnosis at age 5 years, producing the best performing models with AUROC ≥ 0.90 and AUPRC ≥ 0.80. Respiratory infection history, antibiotic exposure, and maternal asthma also continued to have an impact. At age 4 years, wheezing status and atopy remained very important, along with antibiotic exposure and respiratory infections.

When we compared feature importance across the 4-year period, we found that several early-life factors, including birth weight, sex, and ethnicity, lost their predictive importance as data from later time points were included. In contrast, certain factors, such as maternal asthma, non-cold wheezing history, lower respiratory tract infections, and antibiotic exposures, demonstrated consistent and meaningful contributions to asthma prediction across time. These findings suggest that a longitudinal approach to modeling asthma risk is crucial, as certain risk factors may not be as informative in isolation or at a single time point. Additionally, our results highlight the importance of considering both genetic and environmental factors in predicting asthma risk, as maternal asthma and early-life respiratory infections and antibiotic exposures consistently emerged as key predictors in our analyses.

## Discussion

Childhood asthma is a chronic respiratory disease that often persists throughout an individual’s lifetime, imposing a significant burden on both patients and healthcare systems. Despite numerous treatment options, there are currently no curative therapies available for asthma, and patients often require ongoing treatment to manage their symptoms and prevent exacerbations. As such, early identification of children at risk of developing asthma is of utmost importance, as this will enhance early prognostication of patients for families. Identifying children at risk of asthma in early childhood may therefore reduce family uncertainty and potentially improve long-term outcomes for patients and their families.

### Identification of early-life pediatric asthma using ML

ML has emerged as a promising tool in various medical settings, owing to its ability to capture complex and non-linear relationships among multiple predictors and their synergistic effects on the target variable. The highly heterogeneous nature of asthma outcomes, which result from the interplay of genetic, environmental, and clinical factors, makes it a prime candidate for ML prediction models. While several studies have utilized ML to predict various clinical outcomes of pediatric asthma, including hospitalization, exacerbation, response to treatment, and remission,^16^ research on the early identification of pediatric asthma through ML predictive modeling using as extensive predictors over time remains scarce.^17^ Consequently, there is a pressing need for further exploration of ML’s potential in predicting early-life asthma risk, which could aid in timely and personalized prognosis by distinguishing between those most likely to have transient vs. persistent symptoms. These findings are consistent with previous studies that have highlighted the value of ML in medical decision-making.^4,16,17^

By leveraging the CHILD study cohort, we have identified a short list of salient features and ML models that offer promising prospects for identifying children at high risk of asthma. Analysis of these features across different time points has revealed that early-life asthma diagnosis risk prediction is feasible but challenging before 1 year of age. However, accurate asthma diagnosis prediction at the age of 5 years is achievable with high sensitivity, specificity, and precision (AUROC > 0.9, AUPRC > 0.8) when clinical information on parental asthma, wheezing, atopy, respiratory infections, and antibiotics usage is available at age 3 years.

### Identification of important predictors for pediatric asthma

Our ML models demonstrate the significance of a short list of established predictors of asthma and are highly consistent with previous research on risk and protective factors while demonstrating the limited predictive benefit of other correlated factors.^18–28^

Among the features that our models identified as most important for predicting asthma were wheezing status, atopic status, parental asthma, history of respiratory infection, and antibiotics usage. Parental (primarily maternal) asthma emerged as the earliest and most consistent predictor of asthma diagnosis at age 5 years in our models, in agreement with established research.^18,19^

While our models revealed an association between maternal psychological stress and childhood asthma, in agreement with previous research,^20,21^ this relationship appeared to be limited to the first year of life. Our findings support the notion that early antibiotic exposure is associated with an increased risk of childhood asthma,^8,22^ and suggest that strategies to mitigate the impacts of antibiotics may be useful. In addition, our models showed that lower respiratory tract illness in early childhood increases the risk of developing asthma, in agreement with previous studies linking early, lower respiratory illness and later-life asthma.^23,24^ Finally, our models supported the protective effect of exclusive breastfeeding and longer gestational age, consistent with previous researches.^25–28^

### Strengths

Our study boasts several notable strengths, including its longitudinal design, which enables investigation through data collected at multiple time points with short intervals. Specifically, we tracked participants from birth up to four years of age to predict physician-diagnosed asthma at the 5-year mark. By collecting data at various time points, we were able to uncover the relative importance of putative predictors across these time intervals, as well as identify trends in asthma predictive capacity using multiple ML algorithms, both individual and ensemble.

Additionally, our study adopted an agnostic approach to evaluate the importance of all 132 variables without any manual intervention during the feature selection process. This approach was facilitated through ML algorithm discovery, which allowed for the automatic identification of important variables without any prior bias or preconceptions. Notably, this methodology achieved high consistency with established factors for impacting asthma prediction while also revealing less well-known factors such as maternal stress, gestational age, and jaundice as significant contributors to asthma prediction.^29^

Lastly, development and testing of ML predictive models is a complex process that requires careful consideration of several critical factors. Our study stands out by meeting all of these criteria, including the use of an adequate sample size, high-quality training data, algorithmic-based selection of input features, cross-validated tuning and validation of ML algorithms, appropriate and careful study design, and continual clinical involvement during ML model development.^4,16,17,30^ By meeting these requirements, our models achieve high performance and provide reliable and accurate predictions, making them highly applicable in clinical settings. Furthermore, our approach not only contributes to the field of asthma prediction but also serves as a valuable blueprint for the development of ML models in other areas of healthcare.

### Limitations

While the present study utilized data from the large-scale observational CHILD Cohort, it is important to recognize that a large proportion of the data used for developing the ML models were derived from gathered questionnaires and clinical assessments completed by parents and clinicians. Despite using rigorous quality control measures to mitigate survey bias, the data may still be susceptible to other forms of bias, including desirability bias, response bias, and recall bias, which can introduce non-objectivity and noise into the data and ultimately lead to reduced predictive performance of the ML models.

To overcome this limitation and potentially enhance the predictive performance of the models, future studies may benefit from the incorporation of objective measurements, such as biological and genetic markers. However, these types of measurements are often associated with high costs, time constraints, and specialized equipment requirements, which may limit their availability to a much smaller subset of individuals and affect the generalizability of the model to the broader population such as our study. Nevertheless, further research is warranted to evaluate the feasibility and potential benefits of including such measurements to improve the predictive performance of the ML models, particularly at earlier stages of asthma development.

## Conclusions

The present study demonstrates the potential of ML models in effectively predicting asthma diagnosis at age 5 years using early-life data from children. Our results indicate that individual models, including Logistic Regression, Random Forest, SVM, and weighted soft voting ensemble models, were effective in predicting asthma diagnosis. Importantly, our findings suggest that physician-diagnosed asthma at age 5 years could be reliably predicted using non-biological and non-genetic data by the age of 3 years, whereas accurate prediction before 1 year of age using existing clinical dataset was challenging. These results have significant implications for early detection and intervention strategies for asthma.

Furthermore, our study identified parental asthma, wheezing, atopy, lower respiratory infections, and antibiotic exposures as the most important and stable predictors for asthma diagnosis at age 5 years, which are established significant risk factors for asthma. These findings reinforce the importance of early-life exposure to these risk factors and suggest their potential long-term implications for asthma development.

To conclude, our study highlights the potential of ML models in predicting asthma diagnosis at age 5 years and emphasizes the significance of early-life risk factors in the development of asthma. These results have implications for the development of targeted prevention and intervention strategies for asthma and call for further investigation into the utility of ML models in predicting other complex health outcomes.

### Supplementary information

Supplementary information

## Data Availability

All the datasets used and analyzed during this study are available in the CHILD Study Cohort Repository and can be obtained through submitting a Concept Proposal via CHILDdb (Please see https://childstudy.ca/for-researchers/data-access/ for details).
