# Supplementary material for: Early prediction of pediatric asthma in the Canadian Healthy Infant Longitudinal Development (CHILD) birth cohort using machine learning
Source: Pediatr Res. 2024 Jan 11;95(7):1818–25. doi: 10.1038/s41390-023-02988-2 (PMC11245385; doi:10.1038/s41390-023-02988-2)
Supplement: Supplementary file 1 — Supplementary information [file 41390_2023_2988_MOESM1_ESM.pdf]

Online Supplement Materials

Time Point	Variables	No. of Variables
At Birth	Anesthetic delivery, Pollentress Father, Weight for age 0m, F10min Mask Ventilation, No of Pregnancy, Prenatal Cardiac Disorder, CESD 18week, Apgar Score 1min, Jaundice Birth, Mom Atopy, Analgesics usage delivery, Smoke Prenatal Maternal, Prenatal Mother Condition, Respiratory Problems Birth, PSS 36week, Prenatal Induced Hypertension, AD Father, Stay Duration Hospital, AD Mother, Apgar Score 5min, Wheeze Father, Prenatal Hypertension, Child Ethnicity, Prenatal Bleeding, PSS 18week, Mode of delivery, Mother Asthma, Mother Caucasian, Dad Atopy, F10min Free Flow Oxygen, Complications Birth, Prenatal Gestational Diabetes, First 10min Measure, Prenatal Infections, Hayfever Mother, F10min Suction, Wheeze Mother, Prenatal Hypotension, F10min Intubation, Prenatal None Conditions, FAllergies Father, Prenatal Other Conditions, CESD 36week, Prenatal Nausea, F10min No Measure Needed, Smoke Prenatal Secondhand, Father Caucasian, F10min Positive Pressure Ventilation, Pollentress Mother, Hayfever Father, FAllergies Mother, Father Asthma, Study Center, Gest Days, F10min Oxygen Mask, Sex, F10min Perineum suction	57
6 Months	Epi Noncold Wheeze 3m, Home New Furnitures 6m, Epi Noncold Wheeze 6m, Wheeze 3m, PSS 6m, Weight for age 3m, BF Status 3m, Noncold Wheeze 6m, Home Presence Smoke 6m, CESD 6m, Noncold Wheeze 3m, Wheeze 6m, Cumulative Wheeze 6m, BF 1m, Home Furry Pets 6m, Cumulative Wheeze 3m, BF Status 6m	17
1 Year	BF 9m, Wheeze 1y, CESD 12m, Recurrent Wheeze 1y, RIfrequency Earlier 12m, Prolonged Expiration 1yCLA, Child Food 1y, Number of AntibioticsCourse 12m, Wheeze 1yCLA, Crackles 1yCLA, Time of AntibioticsUsage 12m, BF 12m, Noncold Wheeze 1y, Cumulative Wheeze 12m, Epi Noncold Wheeze 1y, PSS 12m, Antibiotics Usage 12m, Child Inhalant 1y, RIseverity Earlier 12m, Child Atopy 1y, Weight for age 12m	21
2 Years	Epi Noncold Wheeze 2y, Wheeze 2yh, Cumulative Wheeze 24m, PSS 24m, Noncold Wheeze 18m, Cumulative Wheeze 18m, BF 24m, CESD 24m, Noncold Wheeze 2y, CESD 18m, BF 18m, Epi Noncold Wheeze 18m, Wheeze 2y, Wheeze 18m, PSS 18m, BF Implied Duration	16
3 Years	Noncold Wheeze 2hy, Noncold Wheeze 3y, Child Food 3y, Epi Noncold Wheeze 2hy, Child Atopy 3y, Child Inhalant 3y, RIseverity Later 36m, Cumulative Wheeze 30m, Cumulative Wheeze 36m, Diastolic BP 3yCLA, Systolic BP 3yCLA, Recurrent Wheeze 3y, Wheeze 3yCLA, Epi Noncold Wheeze 3y, Wheeze 3y, Pulse Rate 3yCLA, RIfrequency Later 36m, Weight for age 36m	18
4 Years	Epi Noncold Wheeze 4y, Wheeze 4y, Noncold Wheeze 4y, Cumulative Wheeze 48m	4

S[illegible]

Abbreviation used in the table. F10min: First 10 minutes after birth delivery; AD: Atopic Dermatitis; CESD: Center for Epidemiologic Studies - Depression; PSS: Psychological Stress Scale; FAllergies: Food Allergies; BF: breastfeeding; Epi represents Number of episodes; CLA: Clinically Assessed by A Physician; BP: Blood Pressure. (A complete list of variables available in the CHILD Cohort Study is available at https://childstudy.ca/for-researchers/study-data/)

Data split	Parent	Caucasian	Other	Hispanic	Black
**Train**	Mother	1184 (79.78%)	229 (15.43%)	41 (2.76%)	30 (2.02%)
	Father	1182 (79.65%)	225 (15.16%)	37 (2.49%)	40 (2.70%)
**Test**	Mother	218 (80.74%)	39 (14.44%)	6 (2.22%)	7 (2.59%)
	Father	219 (81.11%)	32 (11.85%)	11 (4.07%)	8 (2.96%)

Supplemental Table 2. Table of Parental Ethnicity. Count and percentage of study participant ethnicities. In the analyses, ethnicity was coded as Caucasian vs. not due to the small sample sizes of Black and Hispanic and the ambiguity of these classes vs. Other.

Sup[illegible]

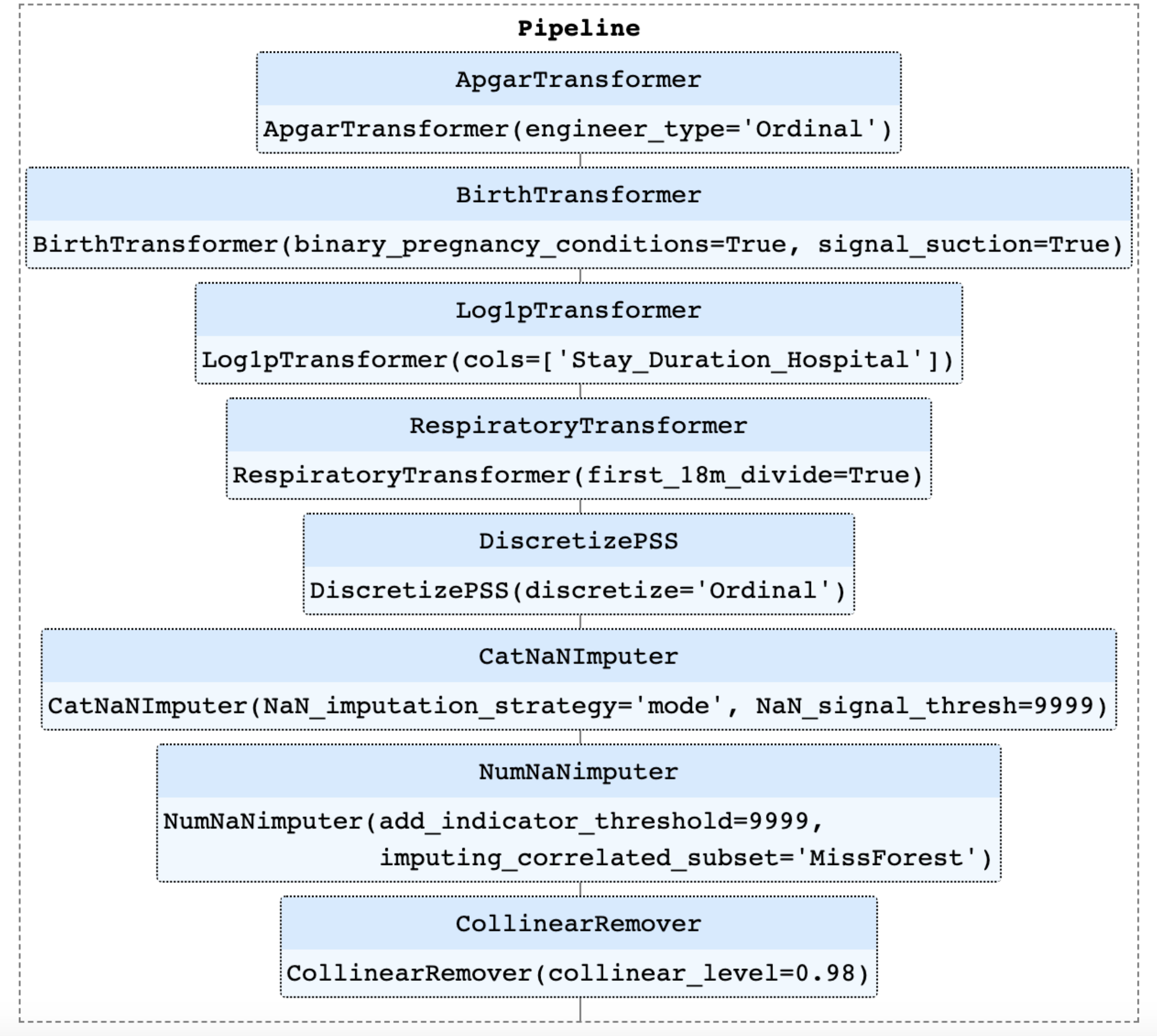

Steps taken to transform the curated data obtained from the CHILD Study Cohort into the ML digestible format. ApgarTransformer was designed to evaluate the effects of different engineering types (“logarithmic”, “categorical”, “original”) for APGAR score evaluated at birth. BirthTransformer was designed to assess the effects of the first 10-minute measures taken immediately after the child is delivered and the mother’s pregnancy conditions.

Log1pTransformer was used to change the distribution of numerical to its log form. DiscretizePSS was used to evaluate the different engineering types for PSS/CESD scoring — a measurement of maternal psychological stress level. CatNaNImputer and NumNaNimputer are used to impute the missingness in the data for categorical and numeric variables. CollinearRemover is to remove highly collinear features in the variable set.

Definition of Key Variables in this study

Target: *Physician-diagnosed Asthma at age 5*

At the clinic visit when the child was 5 years old, spirometry was performed with the ndd Easy on-PC system (ndd Medical Technologies, Inc, Andover, Mass).25 FEV1, forced vital capacity (FVC), and FEV1/FVC ratio were measured and converted to z scores following Global Lung Initiative reference equations, which consider age, sex, height, and ethnicity. Specialist-physician diagnosis remains the criterion standard in pediatric asthma. To reach this diagnosis, each child was assessed through a structured interview with his or her caregivers to identify symptoms consistent with asthma (i.e., recurrent wheeze and coughing without a cold) based on the validated International Study of Asthma and Allergy in Children (ISAAC) questionnaire. At 5 years, study participants were seen by a Royal College of Physicians and Surgeons of Canada–certified paediatric allergist/immunologist or paediatric respirologist or designate thereof.[1]

On the basis of this assessment integrating history and physical examination, as well as evidence of bronchodilator response (i.e. > 12% change in Forced Expiratory Volume in 1 sec), participants were classified as definitely having asthma, possibly having asthma, or not having

asthma, where possible asthma was labelled for either consistent symptoms but no bronchodilator response or no clinical history of symptoms but with bronchodilator response. In this study we only used definite and non asthmatic cases to evaluate the longitudinal importance of predictors for differentiating the positive from non-asthmatic cases.

Input Variable: *Child wheeze*

Information on child wheezing was collected through repeated questionnaires completed by parents at 8 time points (3, 6, 12, 18, 24, 36, 48, and 60 months after the child's birth) and from clinical assessments performed when the children were 1, 3, and 5 years of age. Parents were asked whether their child had a wheezing noise coming from his or her chest either with or without a cold in the past 3, 6, or 12 months. At the clinical visit, parents were again asked if the child had a wheeze in the past 12 months. If the responses to either of these questions was yes, wheezing was recorded as present.[2]

Input Variable: *Child atopy*

Child allergic sensitization is determined through allergy skin test: At the 1-, 3-, and 5-year clinic visits children underwent skin prick testing to a panel of 4 food allergens and 6 (at 1 year) or 10 (at 3 years) or 13 (at 5 years) common aeroallergens. Food allergens tested were peanut, whole cow's milk, egg white, and soybean. At 1 year, inhalant allergens tested included Alternaria tenuis, cat hair, dog epithelium, house dust mites (Der p and Der f) and cockroach. Additional inhalants, namely Cladosporium, Penicillium, Aspergillus fumigatus as well as tree, grass and ragweed pollen mixes, were tested only at 3 and 5 years. A wheal diameter ≥2mm greater than

the negative control was considered a positive skin test, and a child was considered atopic if he or she had ≥1 positive skin test.

Input Variable: *Child respiratory infections*

Information on lower respiratory tract infections (LRTIs) was collected through repeated questionnaires at 3, 6, 12, 18, 24, 30, 36, 48, and 60 months after each child's birth. Having an LRTI was defined as having a cold and fever, as well as any occurrence of cough, chest congestion, and trouble breathing.

Severity information was also collected, where definition of severity was based on reported unscheduled doctor visits, emergency room visits and hospitalizations due to bad cold, fever, chest infection, and coughing episode.

Mild: No doctor visits, Emergence Room (ER) visits or hospitalizations required.

Moderate: Unscheduled doctor visit required due to reasons listed above.

Severe: ER visit or hospitalization required due to reasons listed above.

Input Variable: *Child antibiotics usage*

Information on antibiotics usage was collected through prescription history before 1 year of age, in which both the number of used antibiotics course and time of first antibiotics (age in days) usage were recorded[3].

References:

1. Subbarao P., et al. The Canadian Healthy Infant Longitudinal Development (CHILD) Study: examining developmental origins of allergy and asthma. *Thorax*.**70(10),** 998-1000 (2015).

2. Dai, R., et al. Wheeze trajectories: Determinants and outcomes in the CHILD Cohort Study. *Journal of Allergy and Clinical Immunology.* **149(6)**: 2153-2165 (2022).

3. Subbarao P., Mandhane P.J., & Sears M.R. Asthma: epidemiology, etiology and risk factors. *Cmaj*. **181(9)**, E181-90 (2009).
